# Predictive high-throughput screening of PEGylated lipids in oligonucleotide-loaded lipid nanoparticles for neuronal gene silencing†

**DOI:** 10.1039/d1na00712b

**Published:** 2022-02-04

**Authors:** Apoorva Sarode, Yuchen Fan, Amy E. Byrnes, Michal Hammel, Greg L. Hura, Yige Fu, Ponien Kou, Chloe Hu, Flora I. Hinz, Jasmine Roberts, Stefan G. Koenig, Karthik Nagapudi, Casper C. Hoogenraad, Tao Chen, Dennis Leung, Chun-Wan Yen

**Affiliations:** Small Molecule Pharmaceutical Sciences, Genentech Inc. 1 DNA Way South San Francisco CA-94080 USA yenc3@gene.com; Department of Neuroscience, Genentech, Inc. South San Francisco CA 94080 USA; Molecular Biophysics and Integrated Bioimaging Division, Lawrence Berkeley National Lab Berkeley CA USA; Chemistry and Biochemistry Department, University of California Santa Cruz Santa Cruz CA USA

## Abstract

Lipid nanoparticles (LNPs) are gaining traction in the field of nucleic acid delivery following the success of two mRNA vaccines against COVID-19. As one of the constituent lipids on LNP surfaces, PEGylated lipids (PEG-lipids) play an important role in defining LNP physicochemical properties and biological interactions. Previous studies indicate that LNP performance is modulated by tuning PEG-lipid parameters including PEG size and architecture, carbon tail type and length, as well as the PEG-lipid molar ratio in LNPs. Owing to these numerous degrees of freedom, a high-throughput approach is necessary to fully understand LNP behavioral trends over a broad range of PEG-lipid variables. To this end, we report a low-volume, automated, high-throughput screening (HTS) workflow for the preparation, characterization, and *in vitro* assessment of LNPs loaded with a therapeutic antisense oligonucleotide (ASO). A library of 54 ASO-LNP formulations with distinct PEG-lipid compositions was prepared using a liquid handling robot and assessed for their physiochemical properties as well as gene silencing efficacy in murine cortical neurons. Our results show that the molar ratio of anionic PEG-lipid in LNPs regulates particle size and PEG-lipid carbon tail length controls ASO-LNP gene silencing activity. ASO-LNPs formulated using PEG-lipids with optimal carbon tail lengths achieved up to 5-fold lower mRNA expression in neurons as compared to naked ASO. Representative ASO-LNP formulations were further characterized using dose–response curves and small-angle X-ray scattering to understand structure–activity relationships. Identified hits were also tested for efficacy in primary murine microglia and were scaled-up using a microfluidic formulation technique, demonstrating a smooth translation of ASO-LNP properties and *in vitro* efficacy. The reported HTS workflow can be used to screen additional multivariate parameters of LNPs with significant time and material savings, therefore guiding the selection and scale-up of optimal formulations for nucleic acid delivery to a variety of cellular targets.

## Abstract 2

Development of a high throughput screening workflow to characterize the impact of unique PEG-lipid parameters on oligonucleotide-loaded lipid nanoparticle structure and function.

## Graphical abstract

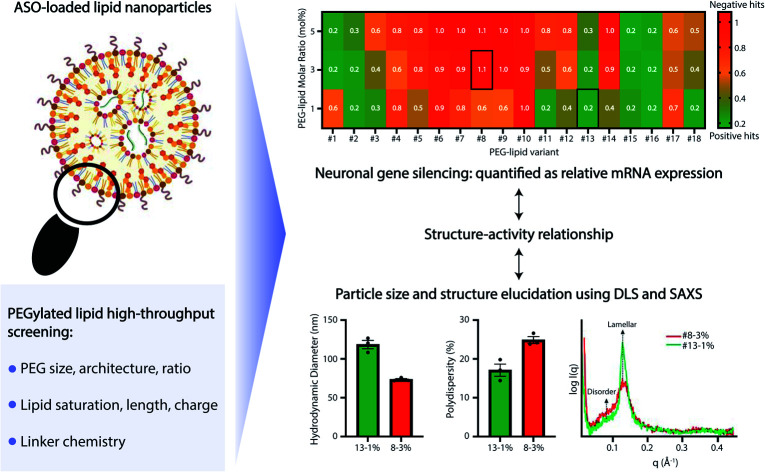

## 1. Introduction

The past few years have seen unprecedented growth in the development of oligonucleotide therapeutics that regulate pathological targets historically deemed undruggable by traditional small molecules and biologics.^1,2^ These therapeutics include aptamers that bind to and alter protein function, short-interfering RNA (siRNA) and microRNA (miRNA) that interfere with coding and non-coding RNA, and antisense oligonucleotides (ASOs) which upregulate or downregulate protein expression, or modulate mRNA splicing in a design-dependent manner.^3–5^ However, several formulation and intracellular delivery challenges need to be addressed to fully realize the therapeutic potential of these molecules.^6–9^

Lipid nanoparticles (LNPs) are one of the most advanced drug delivery platforms that can overcome the challenges associated with oligonucleotide delivery. LNPs not only provide a stable matrix to protect the encapsulated nucleic acid cargo from *in vivo* degradation, but they also improve therapeutic efficacy by enhancing intracellular transport.^10^ The clinical potential of LNP therapeutics was established in 2018 upon U.S. Food and Drug Administration (FDA) approval of the first RNAi-LNP formulation, ONPATTRO®.^11^ More recently, LNP-mediated delivery was utilized in two mRNA vaccines in response to the SARS-CoV-2 (COVID-19) pandemic.^12,13^ In addition to these FDA-authorized therapeutics, there has been a dramatic increase in both clinical and preclinical LNP formulation development.^14,15^ Among these, LNPs are being explored for central nervous system (CNS) targeting, owing to the global burden caused by neurodegenerative diseases and glioma.^16^ Preliminary *in vitro* studies show that pathological genes can be silenced in neurons using LNP-based nucleotide formulations, and ongoing investigations are focused on developing novel LNP compositions that enhance blood–brain barrier penetration to ultimately achieve enhanced *in vivo* efficacy.^17–20^

LNP structure and nucleic acid cargo delivery are regulated by four major components: ionizable lipids, helper phospholipids, cholesterol, and polyethylene glycol-lipids (PEG-lipids).^21^ Cationic ionizable lipids promote the encapsulation of negatively charged nucleic acids during LNP assembly and aid in cytosolic delivery of cargo at endosomal pH (5.5–6.5).^22–27^ Helper phospholipids and cholesterol increase LNP structural stability, promote membrane fusion, and enhance endosomal escape.^23,28–30^ The impact of PEG-lipids is multi-faceted and contributes to what has been termed the ‘PEG-dilemma’.^31^ PEG-lipids control particle size distributions during the self-assembly of LNPs and prevent aggregation.^32–35^ PEG also prolongs *in vivo* LNP circulation time by acting as a steric barrier to the adsorption of plasma proteins.^22,36–38^ While extending half-life increases therapeutic exposure, the hydrophilic PEG corona may also hinder interactions between the particle surface and the lipophilic cell membrane, resulting in poor cellular internalization.^37^ Furthermore, PEG can block transport protein binding, which is essential for LNP cellular internalization *via* receptor-mediated endocytosis.^39^ To overcome the PEG dilemma and achieve optimal intracellular delivery, it is necessary to understand how PEG-lipid characteristics regulate LNP size, structure, and subsequent cargo delivery. Previous studies show LNP efficacy is modulated by several PEG-lipid variables including PEG size, architecture, molar ratio, carbon tail type, and length.^33–36,38,40–47^ However, most published studies focus on a limited set of PEG variables on account of the extensive resources required for manual formulation workflows. Due to the wide screening space for PEG-lipids, a comprehensive study encompassing multiple PEG-lipid types and variables is necessary to empirically understand how PEG-lipids impact LNP structure and function.

An automated high-throughput screening (HTS) workflow to prepare and characterize LNPs can be used to comprehensively assess how PEG-lipid parameters regulate LNP function by allowing complex LNP screening designs, while saving substantial time and materials.^32^ In this study, we generated a library of 54 ASO-LNPs using 18 different PEG-lipids across the phosphoglyceride, diglyceride, and ceramide families, at varying PEG-lipid molar ratios (1, 3, and 5 mol%) using a high-throughput workflow. The impact of PEG-lipid parameters – molecular weight (M.W.), carbon-tail length, and molar ratios – on ASO-LNP size and polydispersity was assessed. Additional PEG-lipid variables including PEG architecture, lipid tail saturation, PEG-lipid charge, and linker chemistry were also analyzed. Following physicochemical characterization, formulations were screened in primary mouse cortical neurons for ASO-mediated regulation of mRNA expression as a function of LNP composition. Target engagement dynamics for representative formulations were evaluated using dose–response curves and these data were correlated to the LNP structures determined by small-angle X-ray scattering (SAXS). In addition, the translatability of ASO-LNP physical properties and *in vitro* performance was validated in primary murine microglia and after scale-up using a microfluidic technique for manufacturing. This study presents the first systematic HTS approach that correlates the impact of PEGylating agents on LNP structure and subsequent *in vitro* ASO delivery efficacy in multiple primary brain cell types. These predictive results support the use of HTS workflows during early-stage development of LNP formulations. This HTS-based formulation approach can also be widely applied to additional LNP screening efforts when identifying optimal formulations for a target cell type or tissue of interest.

## 2. Materials and methods

### 2.1 High-throughput preparation and characterization of ASO-loaded LNPs

Cholesterol, 1,2-distearoyl-*sn-glycero*-3-phosphocholine (DSPC), and all linear PEGylated lipids for LNP formulations were purchased from Avanti Polar Lipids (AL, USA). Branched PEGylated lipid *N*-[2′,3′-bis(methylpolyoxyethyleneoxy)propane-1′-oxycarbonyl]-1,2-distearoyl-*sn-glycero*-3-phosphoethanolamine (DSPE-2arm-PEG-2k) was commercially sourced from NOF America Corporation (NY, USA). The PEGylated lipids screened in this study are listed in Table 1. The ionizable lipid dilinoleylmethyl-4-dimethylaminobutyrate (DLin-MC3-DMA; MC3) was purchased from MedChemExpress (NJ, USA). A murine neuron-targeting 17-mer ASO (M.W. 5635 g mol^−1^, Na-salt form) with phosphorothioate backbone was custom synthesized by BioSpring GmbH (Frankfurt, Germany) using solid-phase synthesis. All other reagents were DNase/RNase-free and used from their commercial sources without further purification.

**Table 1:** PEGylated lipid analogs used for the high-throughput preparation of the ASO-LNP library

PEG lipid #	PEG-lipid name	Lipid family	Charge	C-tail length	C-tail saturation	PEG size (Da)	PEG architecture
1	DMPE (C14:0)-PEG-0.55k	Phosphoglyceride-PE	Negative	14	Saturated	550	Linear
2	DMPE (C14:0)-PEG-1k	Phosphoglyceride-PE	Negative	14	Saturated	1000	Linear
3	DMPE (C14:0)-PEG-2k	Phosphoglyceride-PE	Negative	14	Saturated	2000	Linear
4	DPPE (C16:0)-PEG-1k	Phosphoglyceride-PE	Negative	16	Saturated	1000	Linear
5	DPPE (C16:0)-PEG-2k	Phosphoglyceride-PE	Negative	16	Saturated	2000	Linear
6	DSPE (C18:0)-PEG-0.55k	Phosphoglyceride-PE	Negative	18	Saturated	550	Linear
7	DSPE (C18:0)-PEG-1k	Phosphoglyceride-PE	Negative	18	Saturated	1000	Linear
8	DSPE (C18:0)-PEG-2k	Phosphoglyceride-PE	Negative	18	Saturated	2000	Linear
9	DSPE (C18:0)-2 arm-PEG-2k	Phosphoglyceride-PE	Negative	18	Saturated	2000-B	Branched
10	DOPE (C18:1)-PEG-0.55k	Phosphoglyceride-PE	Negative	18	Unsaturated	550	Linear
11	DOPE (C18:1)-PEG-1k	Phosphoglyceride-PE	Negative	18	Unsaturated	1000	Linear
12	DOPE (C18:1)-PEG-2k	Phosphoglyceride-PE	Negative	18	Unsaturated	2000	Linear
13	DMG (C14:0)-PEG-2k	Diglyceride	Neutral	14	Saturated	2000	Linear
14	DSG (C18:0)-PEG-2k	Diglyceride	Neutral	18	Saturated	2000	Linear
15	Ceramide (C8)-PEG-0.75k	Ceramide	Neutral	8	Saturated	750	Linear
16	Ceramide (C8)-PEG-2k	Ceramide	Neutral	8	Saturated	2000	Linear
17	Ceramide (C16)-PEG-0.75k	Ceramide	Neutral	16	Saturated	750	Linear
18	Ceramide (C16)-PEG-2k	Ceramide	Neutral	16	Saturated	2000	Linear

LNPs with various PEGylated lipids were prepared using a high-throughput approach reported previously.^32^ Briefly, the ASO was dissolved at 93.9 μg mL^−1^ in citrate buffer (25 mM, pH 4.0) and dispensed at 150 μL per well in a 96-well plate (Greiner Bio One 655101, NC, USA) using a TECAN Freedom EVO robotic liquid handler (Tecan Life Sciences, NC, USA). Using the automation setup, different lipid mixtures composed of MC3, DSPC, cholesterol, and respective PEG-lipid analogs were prepared in ethanol at a molar ratio of 40 : 10 : (50 − *X*) : *X*, where *X* = 1, 3, or 5; and a total lipid concentration of 4 mM was used to maintain N : P = 2 for the resulting ASO-LNPs. N : P is defined as the molar ratio of positively-chargeable amine (N) groups in the ionizable lipid to negatively-charged phosphate (P) groups on the nucleic acid backbone. The lipid mixtures were prepared in a 12-channel reservoir plate (Axygen RES-MW12-LP or -HP, NC, USA), and 50 μL of the lipid phases were injected into the ASO solutions in the 96-well plate using the robot (speed = 0.5 mL s^−1^, followed by 10 cycles of mixing with 0.1 mL volume per cycle), resulting in 1 mM total lipids per well at the ethanol : aqueous phase volume ratio of 1 : 3. Each LNP formulation was prepared in triplicate. The ASO-loaded LNPs were diluted in phosphate-buffered saline (PBS, pH 7.4) to achieve a final concentration of 1 μM total ASO per well. The free ASO amounts were measured using a size exclusion (SEC) high-performance liquid chromatography (HPLC) system (Agilent Technologies, Santa Clara, CA) with Tosoh TSKGel UP-SW2000 (4.6 mm × 150 mm dimension, particle size of 2.0 μm, and pore size of 12.5 nm). The total ASO payload in each formulation was quantified using Quant-iT™ Oligreen™ ssDNA assay (Thermo Fisher Scientific) following LNP disruption at 37 °C using 0.2% RNase-free Triton™ X-100 (MilliporeSigma, St. Louis, MO). The determined free and total ASO concentrations were used to calculate the encapsulation efficiency (EE%) for the identified hit formulations. Small aliquots of each sample were transferred into a glass-bottom 96-well plate (Greiner Bio-One 655892, NC, USA) and further diluted 50× in PBS for characterization of their particle size distributions by dynamic light scattering (DLS) using a DynaPro plate reader III (Wyatt Technology, CA, USA).

### 2.2 Animals

Timed pregnant C57BL/6N mice, dams, and pups from Charles River Laboratories (MA, USA) were used to prepare dissociated neuronal and microglial cultures. Upon delivery, a controlled 14 : 10 hour light : dark cycle was maintained, and animals were given unrestricted access to food and water. All animal studies were conducted in accordance with local regulations and the NRC Guide for the Care and Use of Laboratory Animals followed at IBioBA-CONICET. Animal studies were authorized and approved by the local as well as Genentech Inc. Institutional Animal Care and Use Committees.

### 2.3 Murine cortical neuron and microglia cell cultures

Mouse embryonic cortical neurons were cultured as described previously.^48^ Briefly, cortices from day 15 C57BL/6N embryos (E15) were dissected and washed thrice with pre-cooled Hank's balanced salt solution (HBSS; Invitrogen, CA, USA). Cortical tissue was incubated for 10 minutes at 37 °C in HBSS supplemented with 0.25% trypsin (Invitrogen) and DNase I (Roche CustomBiotech, IN, USA). Tissue was washed thrice with cold HBSS and triturated in plating media containing DNAse I (Gibco Neurobasal medium (Thermo Fisher Scientific, MA, USA), 20% heat-inactivated horse serum (Thermo Fisher Scientific), 25 mM sucrose, and 0.25% Gibco GlutaMAX (Thermo Fisher Scientific)). Dissociated cells were centrifuged at 125*g* for 5 minutes at 4 °C. Cells were resuspended in a plating medium and plated in 96-well, poly-l-lysine-coated (PLL; Sigma-Aldrich, MO, USA) plates. After 24 hours, the plating medium was replaced with Neurobasal medium supplemented with 1% B-27 (Thermo Fisher Scientific) and 0.25% Gibco GlutaMAX. Cells were maintained in an incubator at 37 °C with 5% CO_2_ and the medium was renewed using 50% exchange every 3–4 days to maintain cell health.

Mouse microglia were cultured as described previously with the following alterations.^49,50^ Cortices from day 3 C57BL/6N pups (P3) were dissected, stripped of meninges, and washed thrice with pre-cooled HBSS (Invitrogen). Cortical tissue was incubated for 10 minutes at 37 °C in Gibco 0.25% trypsin–EDTA (Thermo Fisher Scientific). Cortices were washed thrice in culture medium (Gibco Dulbecco's Modified Eagle Medium (DMEM), high glucose supplemented with 10% fetal bovine serum and 1% penicillin–streptomycin (Thermo Fisher Scientific)) and triturated. The dissociated cells were 70 μm strained and the cell suspension was centrifuged at 700*g* for 5 minutes at room temperature. The cell pellet was resuspended in the culture medium and plated in PLL-coated 225 cm^2^ flasks at a density of 8 cortices per flask. Cultures were maintained at 37 °C with 5% CO_2_. After 4 days, the attached cells were washed thrice with PBS and a fresh culture medium was added. Confluent astrocyte cultures containing suspended microglia were agitated on a rotary shaker at 200 rpm for 2 hours at 37 °C with 5% CO_2_ after an additional 7 days. The resulting microglia cell suspension was centrifuged at 700*g* for 5 minutes at room temperature. Cells were resuspended in culture medium, counted, and plated in 96-well PLL-coated plates (Sigma-Aldrich).

### 2.4 Cell treatments and RT-qPCR gene knockdown analysis

Murine cortical neurons or microglia were plated at a density of 3 × 10^5^ cells per mL in 96-well plates. PBS, ASO, or ASO-loaded LNPs were added to the cortical neuron and microglia cultures at DIV6–8 and DIV1, respectively, at the concentrations indicated below and were incubated for 24 hours at 37 °C with 5% CO_2_. HTS samples were diluted to 1 μM ASO in PBS, then added directly to the neuron or microglia cell culture medium to achieve a final treatment dose of 50 nM ASO per well. Alternatively, ASO or ASO-LNPs were pre-diluted in neuronal medium to twice the desired final concentration, and 50% media exchange was performed to achieve the desired dosing range for the EC_50_ study. Neurons were treated with free ASO at final concentrations of 1, 10, 50, 250, 1000, 5000, and 10 000 nM to analyze gymnosis and ASO-LNPs were added at 0.2, 1, 5, 25, 50, and 100 nM (final) ASO concentration.

Gene expression was analyzed after treatment for 24 hours using the TaqMan™ Fast Advanced Cells-to-CT™ Kit (Invitrogen, Thermo Fisher Scientific) following the manufacturer's instructions. Briefly, neuronal or microglial cultures were washed with PBS and lysed in the presence of DNase I. The lysis reactions were stopped and reverse transcription was performed at 37 °C for 30 minutes followed by a 95 °C 5 minutes inactivation using a Veriti™ 96-well Fast Thermal Cycler (Applied Biosystems, Thermo Fisher Scientific). Forty cycles of quantitative PCR (qPCR) were conducted at 95 °C for 1 s and 60 °C for 20 s using either a QuantStudio Flex or a ViiA 7 real-time PCR system (Applied Biosystems, Thermo Fisher Scientific). A FAM-MGB mouse probe targeting a neuronal/microglial gene of interest was used to quantify ASO-mediated mRNA knockdown efficiency. Gene expression values were calculated using the comparative delta delta *C*_*t*_ (−ΔΔ*C*_*t*_) method using housekeeper gene (NeuN, neurons or CSF1R, microglia) and PBS-treated *C*_*t*_ values for normalization. VIC-MGB mouse probes for *Rbfox3*/NeuN (Mm01248771_m1) and *Csf1r* (Mm01266652_m1) were obtained from Life Technologies, Thermo Fisher Scientific.

### 2.5 Small-angle X-ray scattering

SAXS data were collected in the high throughput mode (HT-SAXS) using the Advanced Light Source SIBYLS beamline 12.3.1 at the Lawrence Berkeley National Laboratory (CA, USA)^51^ on representative ASO-LNP formulation hits at a total lipid concentration of 1 mM. X-ray wavelength was set at *λ* = 1.216 Å, and the sample-to-detector distance was 2070 mm, resulting in a scattering vector, *q*, ranging from 0.01 Å^−1^ to 0.45 Å^−1^. The scattering vector is defined as *q* = 4π sin *θ*/*λ*, where 2*θ* is the scattering angle. Experiments were performed at 20 °C as described elsewhere.^52^ Briefly, the sample was exposed for 10 s with the detector framing at 0.3 s to maximize the signal while merging the SAXS signal using the SAXS FrameSlice application (https://bl1231.als.lbl.gov/ran). No radiation damage was observed during the 10 s exposure, and all collected frames were merged. The merged SAXS profile was further processed using the SCÅTTER package (https://www.bioisis.net/tutorials) and plotted using the OriginPro 2015 (OriginLab Corporation, MA, USA).

### 2.6 Microfluidic preparation and characterization of ASO-loaded LNPs

A microfluidic mixing method was used for the scale-up preparation of selected ASO-LNP formulations that were identified as either positive or negative hits by the HTS approach.^32^ Lipid mixtures that replicate the selected LNP compositions from HTS were manually prepared and dissolved in ethanol at a total lipid concentration of 4 mM. The ethanol stream was rapidly mixed with an aqueous stream containing 93.9 μg mL^−1^ ASO dissolved in citrate buffer (25 mM, pH 4.0) using a microfluidic laminar mixing device (NanoAssemblr™ Benchtop, Precision NanoSystems, BC, Canada) at a 1 : 3 volume ratio, and a total flow rate of 12 mL min^−1^. The formulated LNPs were purified by centrifugal ultrafiltration (MWCO 10 kDa; Amicon, MilliporeSigma, MA, USA) at 2000*g* for 30 min to remove free ASO and lipids, followed by buffer exchange to RNase-free PBS. Purified formulations were analyzed for their particle size distributions using DLS and ASO payload concentration as described in Section 2.1.

### 2.7 Statistical analysis

Data plotting and statistical analysis were performed using Prism 9.2.0 (GraphPad Software, San Diego, CA). All results are presented as mean ± SEM, *n* = 3 containing 3 averaged internal replicates. To determine EC_50_ values for ASO dose–response treatments, data were analyzed using non-linear regression, and curves were fitted using log (inhibitor concentration) *vs.* response, variable slope (four parameters) equations. The impact of various PEG-lipid parameters on ASO-LNP delivery and subsequent mRNA expression was analyzed using a non-parametric Kruskal–Wallis test followed by *post hoc* comparisons using Dunn's test, at a 95% confidence interval (*p* < 0.05). Linear regression analyses on the particle size datasets were performed using Microsoft® Excel 16.39. Mann–Whitney test was used at the 95% confidence interval (*p* < 0.05) to compare the average hydrodynamic diameters of the best and worst 10% LNP hits identified based on their *in vitro* efficacies.

## 3. Results and discussion

### 3.1 Formulation of ASO-LNP library with diverse PEG-lipids

**Fig. 1:**
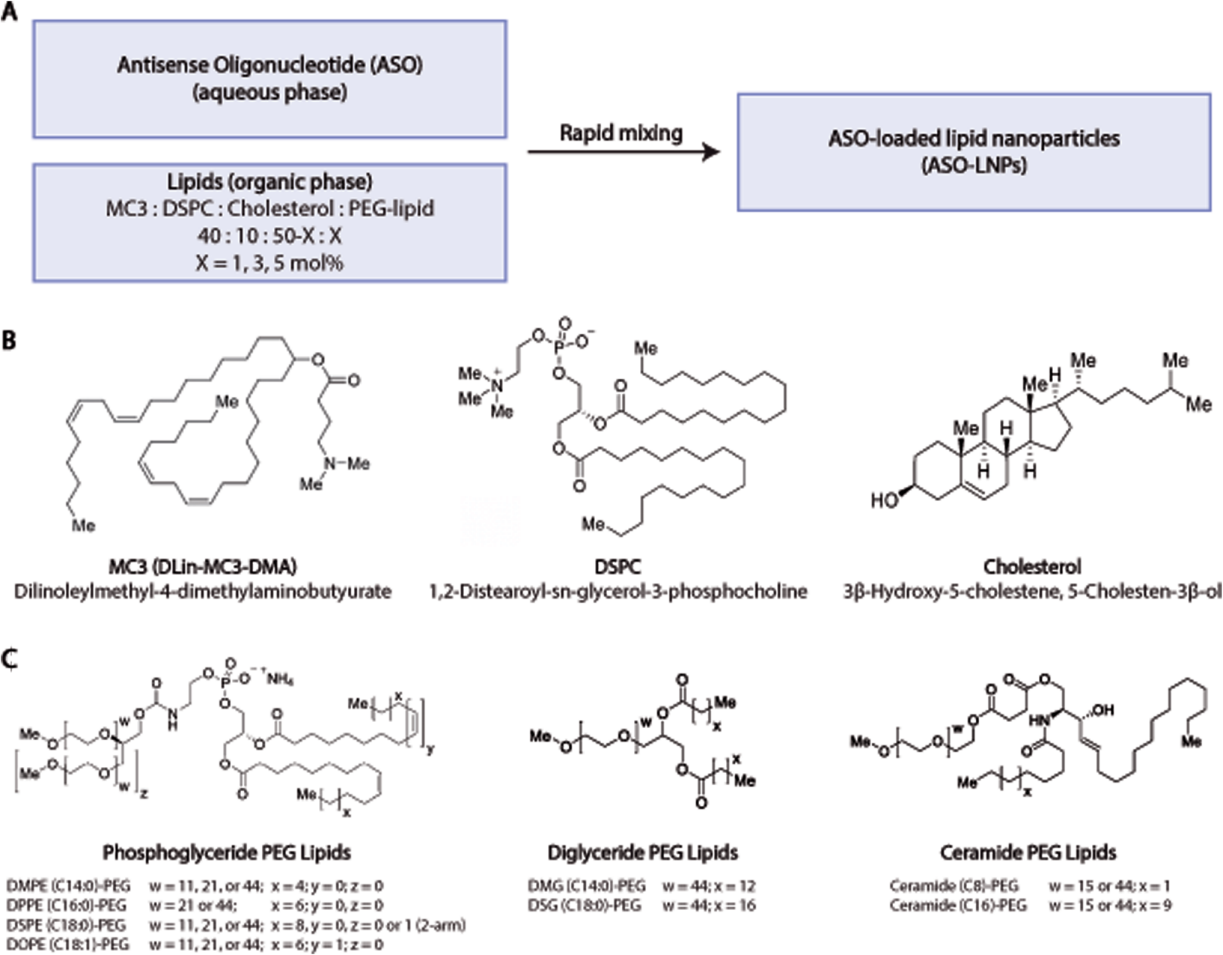
ASO-LNP formulation library. (A) Lipid nanoparticles were prepared using a liquid handling robot by rapidly mixing an aqueous phase containing ASO with an ethanol phase containing dissolved lipid mixtures with varying PEG-lipid compositions. (B and C) Each lipid mixture comprised of the ionizable lipid MC3, helper lipid DSPC, and cholesterol, in combination with a distinct PEG-lipid selected from the phosphoglyceride-PE, diglyceride, or ceramide families to generate an ASO-LNP library of 54 unique formulations.

In our HTS lipid library design, DLin-MC3-DMA (MC3), DSPC, and cholesterol were chosen as the constituent lipids for all ASO-LNP formulations, while the PEG-lipid type and content were varied (Fig. 1A). The combination of MC3, DSPC, and cholesterol (Fig. 1B) is used in ONPATTRO®, the first FDA-approved siRNA-LNP formulation,^53–55^ and is also a benchmark for oligonucleotide delivery in many preclinical LNP model systems.^25,56,57^ To systematically understand the effect of PEG-lipid properties on LNP cargo delivery, several PEG-lipid analogs were incorporated into the ASO-LNPs (Table 1). PEG-lipids commonly used in drug delivery applications were chosen from anionic phosphoglycerides, as well as neutral diglycerides and ceramides.^58,59^ While all of these lipid families are biologically relevant, their chemistries differ significantly. Both phosphoglycerides and diglycerides are composed of fatty acids attached to a glycerol backbone with or without a terminal phosphatidyl ester group, respectively.^60^ In contrast, ceramide lipids contain fatty acids conjugated to a sphingoid base through an amide bond.^61^ These lipid families also have distinct linker chemistries for conjugation of the PEG moiety to the lipidic anchor. Typically, PEG is conjugated to phosphoglyceride lipids *via* a carbamate linkage resulting in a net negative charge on the phosphate group at physiological pH.^47^ Diglyceride and ceramide lipid anchors are conjugated to PEG *via* ether and ester linkages, respectively.^47,62^ In order to understand the impact of linker chemistry on ASO-LNP structure and function, multiple analogs from each of these PEG-lipid families were included while also varying C-tail anchor lengths and/or PEG chain sizes (Fig. 1C). The impacts of PEG architecture (linear or branched) and PEG-lipid C-tail saturation were also assessed.

In addition to investigating different PEG-lipid species, PEG-lipid molar ratio with respect to the total lipid content in the formulation was also varied. The molar ratio of PEG-lipids was tuned to 1, 3, or 5 mol% by adjusting the molar ratio of cholesterol in the lipid mixture.^63^ Using these PEG-lipid parameters, a library of 54 distinct ASO-LNP formulations was prepared using a 96-well plate-based HTS workflow for physicochemical characterization and *in vitro* efficacy evaluation.

### 3.2 PEG content governs ASO-LNP particle size distribution

**Fig. 2:**
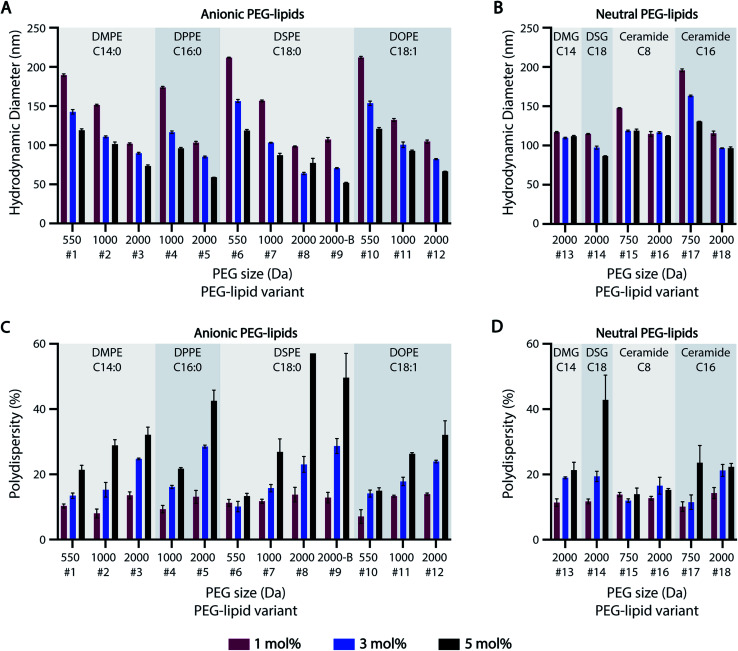
Particle size distribution of ASO-LNP formulations. (A and B) Particle sizes and (C and D) polydispersities of ASO-LNPs containing different subsets of anionic or neutral PEG-lipids, as determined by DLS measurements.

Previous studies suggest that PEG-lipids play an important role in particle size regulation during self-assembly.^32–35^ For the HTS-generated ASO-LNPs, DLS measurements generally indicated there is a negative correlation between particle sizes and molar ratios of PEGylated lipids in the LNP formulations, with mean hydrodynamic diameters ranging between 50–220 nm across the library (Fig. 2A and B). LNPs with short PEG-lipids (PEG M.W. <1000 Da) formulated at 1 mol%, *e.g.* #6 and #10, had the largest particle diameter of 212 nm. In contrast, LNPs containing 5 mol% of long PEG-lipids generally produced small nanoparticles. For example, PEG-lipid #9 showed the smallest mean particle diameter of 52 nm at 5 mol%. This trend is likely due to increased steric hindrance by the PEG chains when they are present at high molar ratios or M.W., which leads to smaller particle sizes by preventing particle growth and aggregation. In general, smaller nanoparticles were formed as PEGylated-lipid molar ratios were increased from 1 to 5%. Specifically, anionic PEG-lipids showed a distinct decrease in particle diameters with increasing PEG sizes and PEG molar ratios in the LNP composition (Fig. 2A). These findings suggest that both the repulsive forces between charged head groups and the steric barrier of PEG chains regulate particle size distributions of LNPs formulated with anionic PEG-lipids. In contrast, LNPs prepared with neutral diglyceride or ceramide PEG-lipids showed only a weak dependence of particle size on PEG content, with no correlation seen for formulations #13 and #16 (Fig. 2B). Additionally, a comparison of linear and branched PEG variants of DSPE-PEG-2k (#8 and #9, respectively) showed no significant effects of PEG architecture on hydrodynamic diameter (Fig. 2A).

While increasing PEG-lipid molar ratio showed charge-dependent effects on particle diameter, PEG-lipid content had an overall positive correlation with LNP polydispersity (% PD) (Fig. 2C and D). Notable exceptions to this trend were #6, #15, and #16. Formulations #15 and #16 contained neutral ceramide-C8 PEG-lipids, which also showed little to no correlation between PEG-lipid content and particle size (Fig. 2B). In agreement with our previous data,^32^ PEG-lipids formulated at 5 mol% with long PEG arms (2000 Da) had high polydispersity, possibly due to the presence of micellar PEG-lipid sub-populations.^64,65^

The effect of lipidic anchor groups with different C-tail features was also analyzed (Fig. 2). For ASO-LNPs formulated with the same molar ratio of PEG-lipids, lipid tail length (DMPE *vs.* DPPE *vs.* DSPE, DSG *vs.* DMG, and Cer-C8 *vs.* Cer-C16), saturation levels (DSPE *vs.* DOPE), and linker chemistries (DMPE *vs.* DMG, and DSPE *vs.* DSG) did not significantly affect LNP sizes or polydispersity indices. These data contrast with the distinct impact of the hydrophilic PEG blocks on LNP properties. Similar observations have been previously reported for siRNA-LNPs prepared with PEG-lipids containing 14, 16, or 18-carbon chains.^36^

Taken together, these data suggest that LNP size distribution is primarily dependent on surface stabilizing PEG content rather than lipid-tail attributes of PEG-lipids, and this PEG-dependence is especially dominant in anionic PEG-lipid scaffolds.

### 3.3 PEG-lipid carbon-tails differentially regulate ASO-LNP *in vitro* efficacy

The global burden from neurodegenerative diseases has contributed to a dramatic increase in brain-targeting therapeutics, including ASOs.^66,67^ While ASOs alter mRNA expression levels or splicing in a design-dependent manner, increasing cellular uptake and delivery efficiency is essential to improve ASO efficacy.^68,69^ To this end, our HTS-generated ASO-LNP library was used to examine how various PEG-lipid parameters affect downstream ASO delivery and efficacy in primary murine cortical neurons. Cortical neurons are a relevant model for many neurodegenerative diseases and enhancing ASO delivery to these historically difficult-to-transfect cells is essential for therapeutic development.

**Fig. 3:**
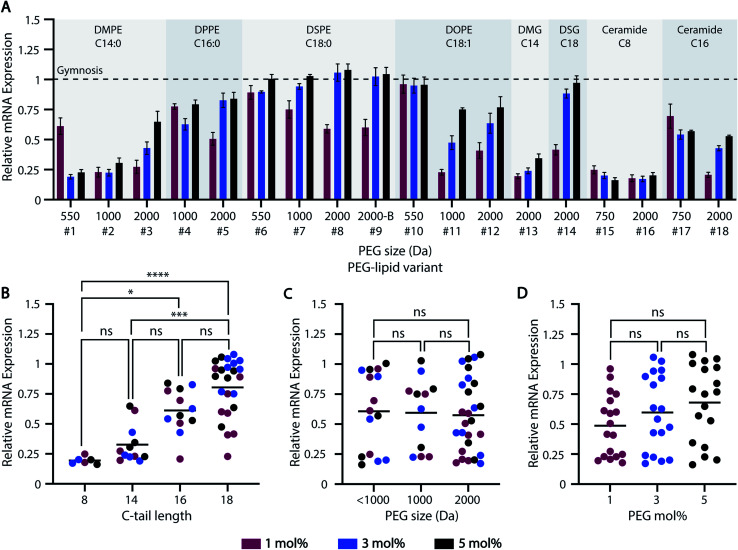
ASO-LNP gene silencing efficacy screening in primary murine cortical neurons. (A) Gene silencing was analyzed using RT-qPCR. Data were normalized to NeuN and PBS-treated controls, and are shown as relative to gymnosis (dotted line). ASO-LNP formulations were re-grouped based on their (B) C-tail length, (C) PEG size, and (D) molar ratio of the PEG-lipids to assess the significance of each parameter on ASO-LNP *in vitro* efficacy (Kruskal–Wallis test and Dunn's test, *p < 0.05, ***p < 0.001, ****p < 0.0001, ns: non-significant).

The majority of ASO-LNP formulations reduced mRNA expression levels in cortical neurons relative to gymnosis (naked ASO) controls, suggesting that encapsulating ASOs in LNPs improves intracellular delivery (Fig. 3A). Among the various PEG-lipid characteristics examined, carbon-tail length played a significant role in determining the extent of mRNA downregulation, with longer carbon chains resulting in a stepwise increase in mRNA expression levels (Fig. 3B). This can be explained by the poor PEG-shedding properties exhibited by PEG-lipids with longer hydrophobic lipid anchors, as extensively demonstrated in previous literature.^34,36,44,70,71^ PEG-lipids with short acyl chains have faster desorption from the LNP surface owing to favorable thermodynamics for cleaving anchoring bonds. This trend was observed for both anionic phosphoglyceride PEG-lipids, as well as neutral diglyceride and ceramide PEG-lipid-based formulations (Fig. S1A†). Comparison of phosphoglyceride PEG-lipids with saturated and unsaturated carbon-tails showed no major impact on mRNA expression with PEG sizes <1000 Da (#6 *vs.* #10). However, mRNA reduction improved for 1000 and 2000 Da PEG-lipid variants when a C=C bond was introduced in the lipid tail (#7 *vs.* #11, #8 *vs.* #12), possibly due to the relatively lower stability of the unsaturated chains allowing for faster PEG-lipid shedding (Fig. S1B†). Furthermore, *in vitro* efficacy was also affected by the PEG-linker chemistry of the PEG-lipids. For example, DMG-C14-PEG-2k (#13) showed a higher reduction in mRNA expression compared to DMPE-(14:0)-PEG-2k (#3), at all molar ratios studied. This result can be attributed to the tailored structure of DMG–PEG facilitating rapid PEG dissociation from the LNP surface, which enhances transfection.^24,72^ Similarly, ceramide-C16-PEG-2k (#18) performed better than the homologous phosphoglyceride PEG-lipid DPPE-(16:0)-PEG-2k (#5). These data are in congruence with previous studies reporting favorable conformational properties of ceramide-PEGs, and their better exchangeability with lipid cell membranes in comparison to their anionic phosphoglyceride counterparts.^42,43,62^ Furthermore, the rapidly hydrolyzing ester linkages between the PEG and ceramide backbone may lead to faster PEG shedding from the LNP surface, as compared with amide-linked PEG in phosphoglycerides.^73^ Thus, these data show that ASO-LNP delivery not only depends on PEG-lipid tail length, but also on the PEG-lipid linker chemistry.

Unlike C-tail length, PEG M.W. and mol% did not have a significant bearing on LNP delivery and ASO-mediated mRNA downregulation (Fig. 3C and D). For example, no major impact was observed across PEG-lipid analogs containing various PEG sizes and 18-C tails formulated at 5 mol% PEG-lipid (Fig. S1C†). Similarly, mRNA expression was not affected by different PEG architectures of the PEG-lipids, *e.g.* linear (#8) *vs.* branched (#9) variants of DSPE-PEG-2k. However, additional lipids with branched PEG arms should be investigated to establish a reliable correlation between PEG architecture and LNP *in vitro* efficacy.

Thus, these HTS data suggest that the gene-silencing efficacy of PEGylated ASO-LNPs strongly depends on their PEG-shedding ability, as governed by the C-tail features and linker chemistry, to allow desirable membrane interactions for intracellular delivery. Future studies will focus on understanding the mechanism of ASO-LNP cellular internalization and endosomal escape that results in ASO-mediated mRNA downregulation.

### 3.4 HTS can be leveraged to identify ASO-LNP behavioral trends

As demonstrated, our HTS approach allows for the quick preparation and characterization of diverse ASO-LNP formulations in a 96-well plate format. This high-throughput workflow can be seamlessly extended to evaluate LNP delivery in additional target cell lines (see Section 3.6). Furthermore, owing to the small working volume and parallel sample handling, our HTS approach leads to significant material and time savings. It also generates robust datasets by directly comparing ASO-LNP formulations in identical environments, thus minimizing processing variations.

In addition to the empirical benefits, our HTS approach also offers data analysis and interpretation advantages. First, physical characterization (Fig. 2) and efficacy data (Fig. 3) involving numerous formulations are generated in short timeframes. Second, comprehensive screening allows for the identification of correlations that may be masked with approaches involving narrow sample sizes. In this study, our HTS datasets showed that hydrophilic blocks of PEG-lipids govern LNP particle size distributions (Fig. 2 and S2†), whereas the hydrophobic lipid tails regulate the cellular interactions necessary for LNP uptake and ASO-mediated mRNA downregulation (Fig. 3). These behavioral trends can also be defined quantitatively using predictive correlations and regression analyses. For instance, linear regression models corroborated the significance of PEG-lipid charge, molar ratio, and PEG size on LNP particle size distributions (*p* < 0.05), as compared to the non-significant impact of different C-tail attributes (Sections S1A and S1B). The correlation accuracy, as represented by *R*^2^ values of 0.869 and 0.700 for anionic and neutral PEG-lipids respectively, can be further improved using iterative screening of wider sample sets, in combination with higher-order curve fittings or advanced machine learning algorithms.^74^

**Fig. 4:**
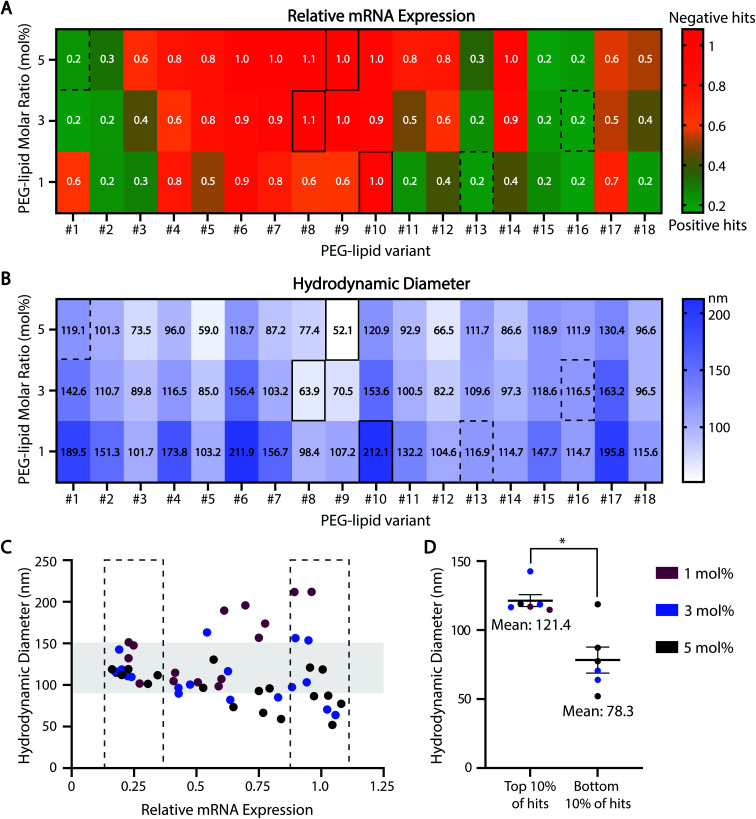
Behavioral trends across the ASO-LNP HTS library. The HTS datasets from Fig. 2 and 3 are presented using color-coded heat maps to rank-order the 54 ASO-LNP formulations based on their *in vitro* efficacies (A) and average particle sizes (B). Representative positive and negative hits are highlighted by dotted and solid rectangles, respectively. (C and D) The correlation between LNP diameter and relative mRNA expression in the top and bottom 10% of ASO-LNP hits suggests an optimal size range of 100–150 nm for efficacious formulations (Mann–Whitney test, *p < 0.05).

Our HTS approach is also useful for rank-ordering ASO-LNP formulations based on mRNA downregulation (Fig. 4A). On a heat map, formulations with relative mRNA expression levels lower or higher than gymnosis are depicted as positive (green) or negative (red) hits, respectively. Correlating ASO-LNP efficacy with the corresponding DLS measurements (Fig. 4B) revealed that a 100–150 nm particle size range was optimal for achieving reduced mRNA levels in cortical neurons (Fig. 4C and D). Formulations within this range can be referenced back to their constituent PEG-lipid features to identify optimal LNP compositions for neuronal delivery. Taken together, these HTS findings suggest that a combination of PEG-lipids with shorter C-tails and lower PEG-lipid mol% in LNPs can result in improved *in vitro* activity, which is in agreement with findings from previous LNP literature.^53,75,76^

### 3.5 ASO-LNP hits exhibit distinct delivery dynamics and structural features

Motivated by the clinical relevance of their constituent PEG-lipids, further characterization of ASO-LNP formulations #13-1% (neutral PEG) and #8-3% (anionic PEG) was performed. Diglyceride DMG(C14)-PEG-2k (PEG-lipid in #13-1%) has been used in many preclinical and clinical LNP formulations, including ONPATTRO®. Thus, there is a mounting interest in understanding the function of this PEG-lipid analog.^53–55^ On the other hand, DSPE(18:0)-PEG-2k (PEG-lipid in #8-3%) has been widely used for nanoparticle surface functionalization, owing to its established clinical performance in DOXIL liposomal formulations.^77^ Formulation #13-1% reduced mRNA expression in cortical neurons by ∼80%, whereas #8-3% showed no significant change when normalized to gymnosis (Fig. 3A). The delivery dynamics and internal structures of these ASO-LNPs were further examined to understand their observed efficacy differences in this study.

**Fig. 5:**
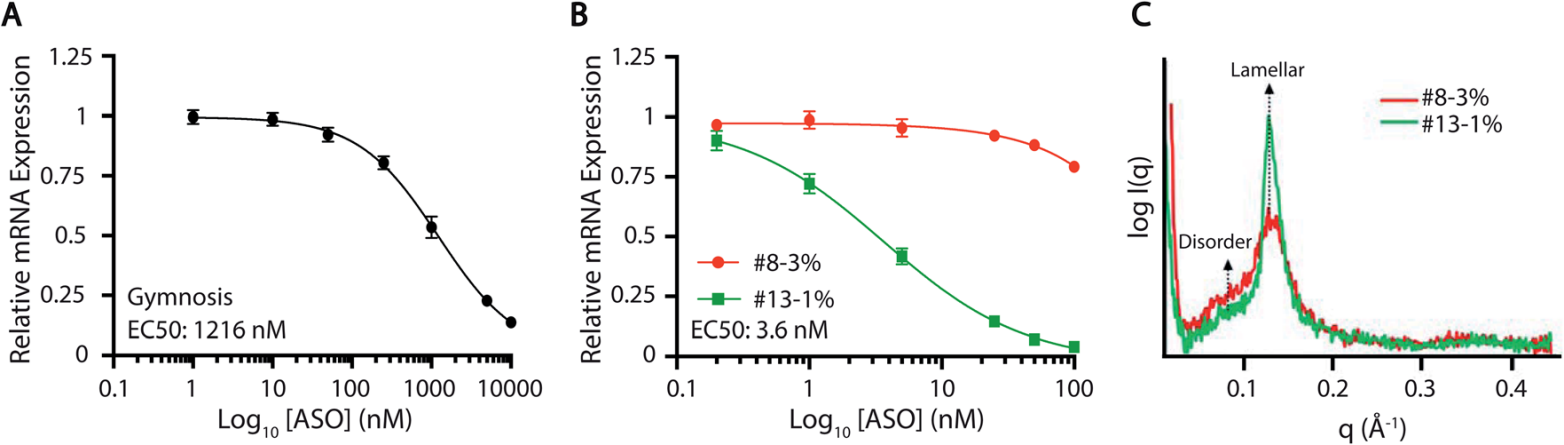
Characterization of representative ASO-LNP positive and negative hits. Dose-response curves were generated in cortical neurons by analyzing relative mRNA expression using RT-qPCR for (A) gymnosis, and (B) positive (green) and negative (red) ASO-LNP hits. Data were normalized to NeuN and PBS-treated control cells. (C) Structural features of the positive (green) and negative (red) ASO-LNP hits were analyzed using small-angle X-ray scattering. The repeat spacing of lipid/ASO/water layer lamellar phase is reflected as the peak maxima at *q* ∼ 0.13 Å^−1^. A lower intensity peak of #8-3% LNP compared to the #13-1% LNP corresponds to smaller periodicity along with the lamellar phase. The broadening of the peaks for #8-3% LNP suggest a disorder of the lamellar phase.

Dose–response curves were generated in cortical neurons to validate the differential activities of ASO-LNPs #13-1% and #8-3%, and the results were compared to gymnosis. Treating cortical neurons with free ASO resulted in an EC_50_ value of 1216 nM (Fig. 5A). In contrast, ASO-LNPs showed varied efficacies based on composition. While the positive hit #13-1% had an EC_50_ value of 3.6 nM, the negative hit #8-3% did not reach a 50% reduction in mRNA expression over the same dosing range (Fig. 5B). Taken together, these data showed that ASO activity is concentration-dependent during gymnosis and LNP delivery. Furthermore, in this cell culture system, LNPs can dramatically and differentially improve ASO delivery as compared to ASO internalized *via* gymnosis, depending on the PEG-lipid used. Further *in vivo* studies are needed to understand the translatability of these *in vitro* efficacy findings.

The representative positive and negative ASO-LNP hits, #13-1% and #8-3%, were further characterized using SAXS to discern the underlying structure–function relationship governing their distinct cellular activities (Fig. 5C). Both LNP formulations showed the presence of one prominent peak in the *q* range of 0.1–0.2 Å^−1^, derived from the repetition of lipid membrane/ASO/water layers in the particle structure.^78^ This is in agreement with previously observed SAXS profiles of cationic lipid–DNA complexes (lipoplexes) coated with PEG-lipids^42^ or siRNA-loaded LNPs.^79,80^ The repeat spacing *d* (thickness of lipid/ASO/water layer) can be quantified from the SAXS profiles as *d* = *2*π/*q*. Thus, the peak positions at *q* = 0.129 Å^−1^ for #13-1% ASO-LNPs and *q* = 0.131 Å^−1^ for #8-3% ASO-LNPs indicate a relatively thicker lamellar layer for the #13-1% formulation as compared to #8-3% formulation (48.7 Å for #13-1% *vs.* 47.8 Å for #8-3%). Moreover, the most significant structural differences between the two formulations can be elucidated through the width and height of their respective SAXS peaks. Lower peak height, as observed for #8-3% ASO-LNPs, correlates to a fewer number of the lamellar layers, and is consistent with the significantly smaller particle size (63.9 nm for #8-3% *vs.* 116.9 nm for #13-1%, Fig. 2A and B). Furthermore, we hypothesize that broadening of the peak and increased signal intensity when *q* < 0.11 Å^−1^ suggests a disordered LNP core structure for #8-3% ASO-LNPs; whereas the #13-1% ASO-LNPs adopt a more ordered lamellar structure that promotes cellular transfection. The absence of the ‘nucleic acid peak’, which is a fingerprint of the lipoplex structure generated by the nucleic acid packed in a hexagonal lattice,^81,82^ suggests that the ASO is not densely packed within the LNPs and may lead to a faster intracellular release of the cargo. Based on these preliminary SAXS analyses, it is hypothesized that the equilibrium between lamellar layers and a disordered core plays an essential role in determining the delivery efficacy of ASO-LNPs. The precise characterization of the structural organization of ASO-LNPs will be the subject of in-depth investigations in the future.

### 3.6 HTS results translate to additional cell culture systems and scaled-up formulations

Our HTS approach, like all screening assays, only becomes valuable if the results can be smoothly translated across additional cell culture systems and scaled-up formulations. Therefore, in order to validate the translatability of our HTS results, three positive hits (#13-1%, #16-3%, #1-5%) and three negative hits (#10-1%, #8-3%, #9-5%) were chosen from the HTS library based on their *in vitro* efficacies. To ensure robust validation of HTS predictability across a broad range of LNP features, the representative formulations included diglyceride (#13), ceramide (#16), and phosphoglyceride (#1) PEG-lipids with different lipid tail saturation levels (#10 *vs.* #8), PEG architectures (#9), and PEG-lipid contents (1, 3, 5 mol%).

**Fig. 6:**
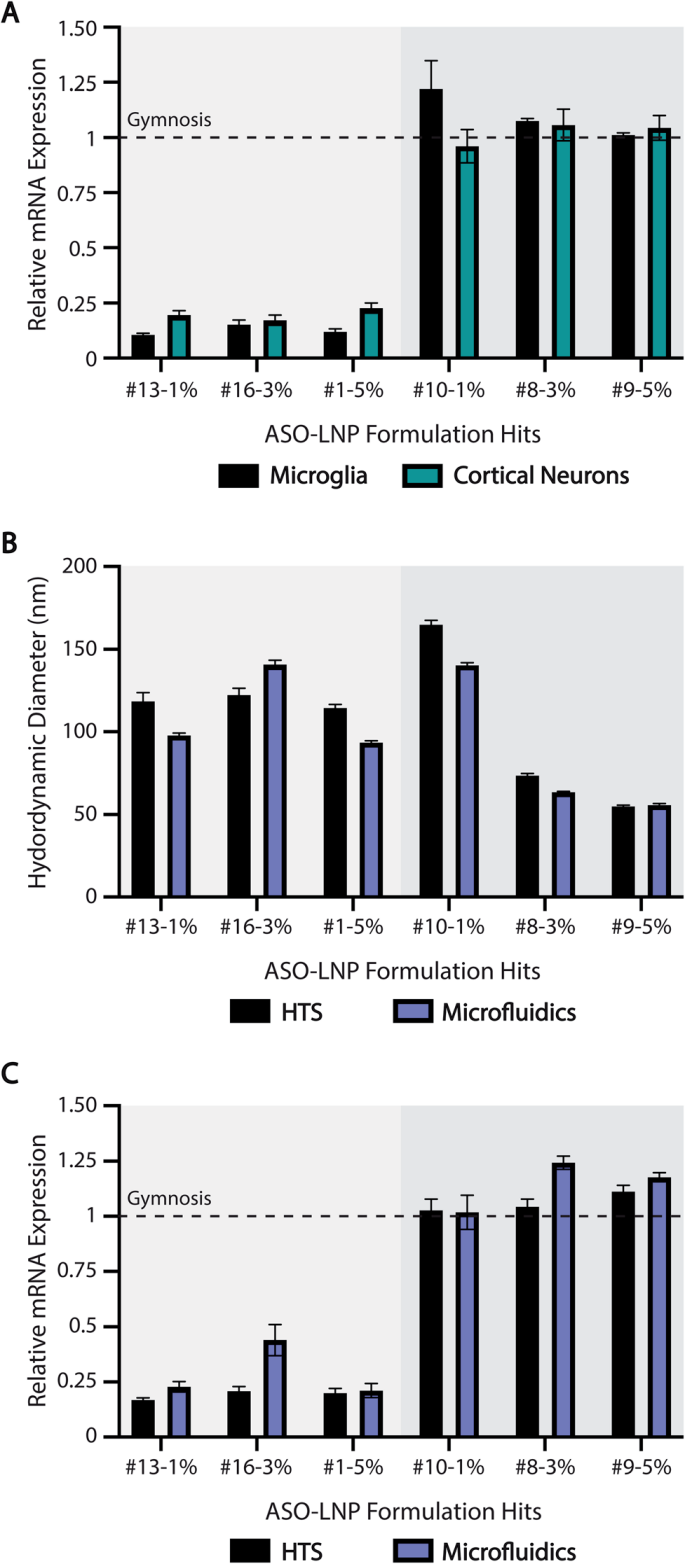
ASO-LNP properties translate across cell types and formulation scales. (A) Selected positive and negative ASO-LNP hits were screened in primary microglia for their gene silencing activity as analyzed using RT-qPCR. Data are normalized to CSF1R and PBS-treated controls, and are shown relative to gymnosis (dotted line). Gene silencing results in cortical neurons were obtained from the HTS experiment shown in Fig. 3. (B) Particle sizes and (C) *in vitro* gene silencing efficacies of the selected ASO-LNPs were compared across the two formulation scales and techniques to validate the translatability of LNP performance. Results are presented as mean ± SEM, *n* = 3 containing 3 averaged internal replicates.

To determine if the results generated in cortical neurons translate across unique cell types and culture systems, the six hit formulations were tested for ASO-mediated mRNA downregulation in murine microglia cultures. Microglia are the tissue-resident macrophages of the brain and are key targets for treating neurodegenerative diseases.^83^ Furthermore, unlike neurons, microglia require serum-containing media to thrive in culture and are highly phagocytic, making these cells a unique comparison to our previous HTS data. Formulations #13-1%, #16-3%, and #1-5% reduced mRNA levels in microglia by ∼80%. In contrast, formulations #10-1%, #8-3%, and #9-5% had no activity (Fig. 6A). These data are consistent with our efficacy screening results in cortical neurons (Fig. 6A and 3) and support our methodology as a broadly applicable screening tool across cell culture systems, including in the presence or absence of serum proteins.

We also studied the translatability of our HTS results to scaled up formulations using a microfluidic mixer commonly used in manufacturing.^80,84,85^ The ASO-LNP hits were scaled up 10× to generate ∼2 mL of each formulation using NanoAssemblr™ Benchtop with the same compositions as their HTS counterparts. DLS characterization showed that the particle sizes were comparable across both formulation techniques (Fig. 6B). ASO encapsulation efficiencies (EE%) of the six hit LNP formulations prepared by HTS and microfluidic methods were comparable with a slight decreasing trend for higher PEG molar ratios (Fig. S3†), which aligns with our previously reported results.^32^ Importantly, the scaled-up ASO-LNP formulations also showed comparable mRNA expression in mouse neurons, relative to their corresponding HTS formulations (Fig. 6C). Irrespective of the preparation technique, positive hit formulations (#13-1%, #16-3%, and #1-5%) showed a 4 to 5-fold reduction in relative mRNA expression, compared to negative hit formulations (#10-1%, #8-3%, and #9-5%). Taken together, these results highlight the reliable and predictive nature of our HTS approach in translating ASO-LNP physical characteristics and *in vitro* efficacies. This translation is especially noteworthy considering the processing differences between the HTS and microfluidics methods such as mixing patterns, downstream processing, final buffer environment, and preparation scale. Despite these differences, the HTS approach reliably predicted ASO-LNP physicochemical properties and cellular efficacy of microfluidics-based formulations. Microfluidics technology has been adapted for use in preclinical and clinical trials due to the ability to scale LNP-based drug delivery vehicles from milliliters to liters. Thus, our predictive HTS approach can be used to screen formulations prior to scale-up, saving significant resources that are typically required for optimizing formulations on the microfluidics platform.

## 4. Conclusions

We designed an HTS approach to understand how ASO-LNP size distribution and cellular delivery vary as a function of PEG-lipid characteristics including PEG size, PEG-lipid content, and carbon-tail structures. The identified ASO-LNP positive hit formulations decreased mRNA expression by 4 to 5-fold in primary cortical neurons and microglia compared to gymnosis. These data demonstrate the translatability of our HTS methodology across unique biologically-relevant cell types and culture systems, including in the presence and the absence of serum proteins. In addition, a smooth translation of size distributions as well as cellular activity between HTS formulations and those prepared using the microfluidic mixing technology was demonstrated, validating HTS as a predictive tool to guide formulation development. By narrowing down hits using HTS, promising formulations can be scaled-up for *in vivo* safety analysis and efficacy profile generation with significant time and material savings. Therefore, with the ability to screen a wide range of LNP parameters and effectively predict *in vitro* performance, this HTS approach represents a promising and cost-effective strategy to rapidly optimize multivariate LNP-based formulations and advance selected candidates during early-stage development.

## Conflicts of interest

There are no conflicts to declare.

## Supplementary Material

NA-004-D1NA00712B-s001
