# Supplementary material for: Predictive high-throughput screening of PEGylated lipids in oligonucleotide-loaded lipid nanoparticles for neuronal gene silencing
Source: Nanoscale Adv. 2022 Feb 4;4(9):2107–23. doi: 10.1039/d1na00712b (PMC9417559; doi:10.1039/d1na00712b)
Supplement: NA-004-D1NA00712B-s001 [file NA-004-D1NA00712B-s001.pdf]

Electronic Supplementary Material (ESI) for Nanoscale Advances.
This journal is © The Royal Society of Chemistry 2022

Supporting Information

Predictive high-throughput screening of PEGylated lipids in oligonucleotide-loaded lipid nanoparticles for neuronal gene silencing

Apoorva Sarode[1,*], Yuchen Fan[1,*], Amy E. Byrnes[2,*], Michal Hammel[3], Greg L. Hura[3,4], Yige Fu[1], Ponien Kou[1], Chloe Hu[1], Flora I. Hinz[2], Jasmine Roberts[2], Stefan G. Koenig[1], Karthik Nagapudi[1], Casper C. Hoogenraad[2], Tao Chen[1], Dennis Leung[1], Chun-Wan Yen[1,†]

[1]Small Molecule Pharmaceutical Sciences, Genentech Inc., 1 DNA Way, South San Francisco, CA-94080, USA

[2] Department of Neuroscience, Genentech, Inc., South San Francisco, CA 94080, USA

[3]Molecular Biophysics and Integrated Bioimaging Division, Lawrence Berkeley National Lab, Berkeley, CA, USA

[4]Chemistry and Biochemistry Department, University of California Santa Cruz, Santa Cruz, CA, USA

[*] These authors contributed equally to this work
[†] Corresponding author, Email: yenc3@gene.com

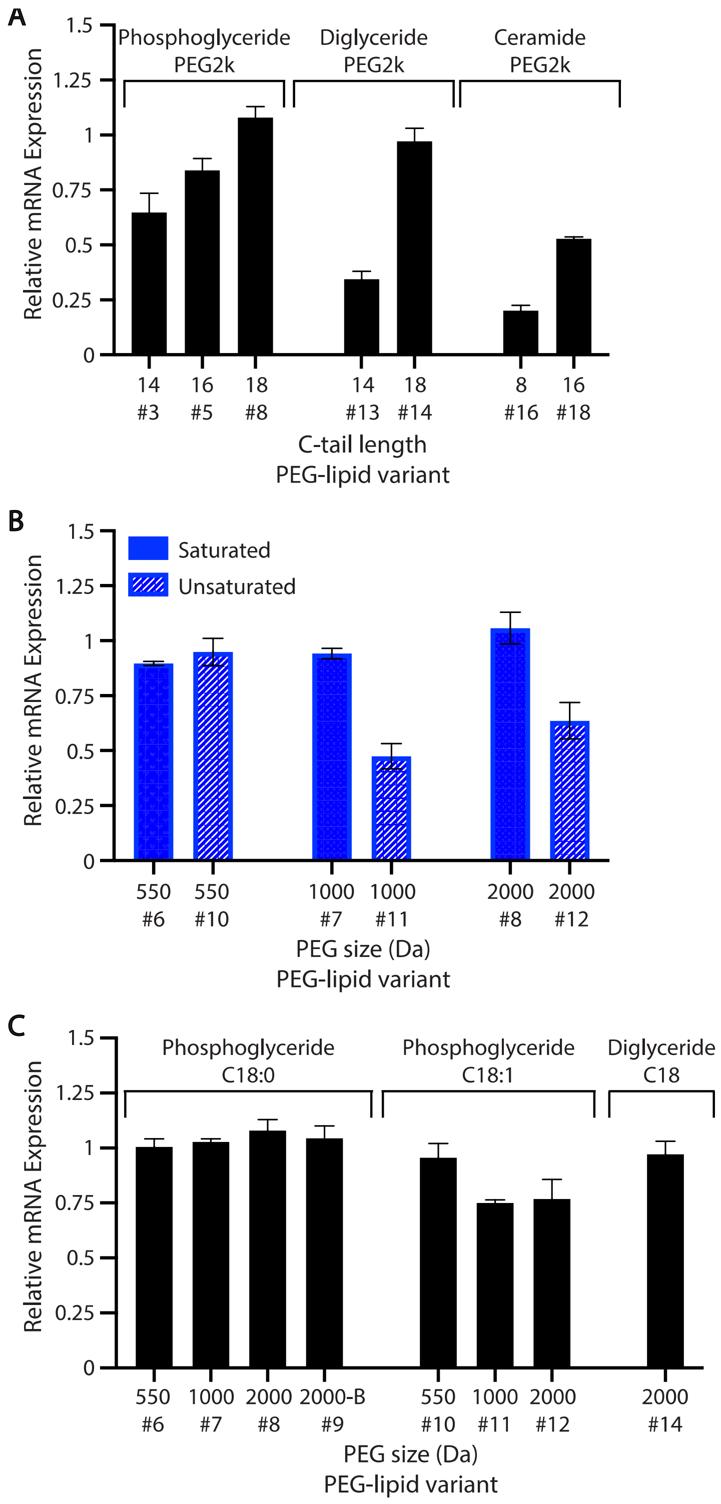

Figure S1. PEG-lipid attributes differentially regulate *in vitro* ASO efficacy. ASO-LNP formulations were prepared using **(A)** 5 mol% PEG-lipids with 2 kDa PEG, but different lengths of their hydrophobic carbon tails, **(B)** 3 mol% C-18 phosphoglyceride PEG-lipids containing saturated or unsaturated carbon tails, and **(C)** 5 mol% PEG-lipids with C-18 lipid tails, but varying sizes of their PEG chains. Results were replotted from the HTS data shown in **Figure 3** and are displayed relative to gymnosis.

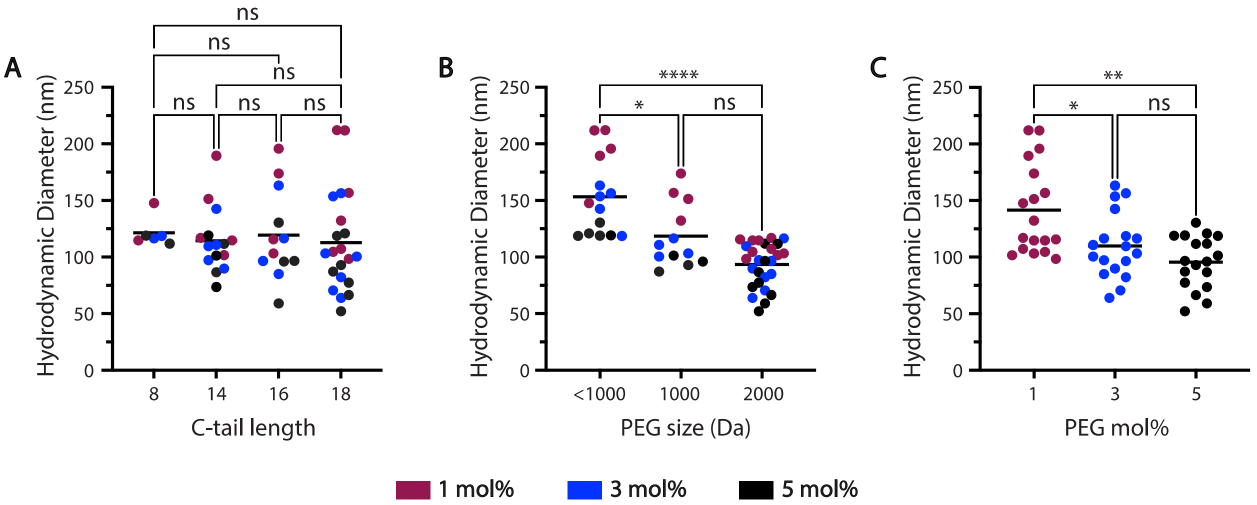

Figure S2. PEG-lipid attributes differentially regulate ASO-LNP particle size. DLS measurements for the ASO-LNP formulations were re-grouped based on **(A)** C-tail length, **(B)** PEG size, and **(C)** PEG-lipid molar ratio in LNPs to assess the significance of each parameter on the average hydrodynamic diameters of the LNPs. (Kruskal-Wallis test and Dunn's test, *$p < 0.05$, **$p < 0.01$, ****$p < 0.0001$, ns: non-significant)

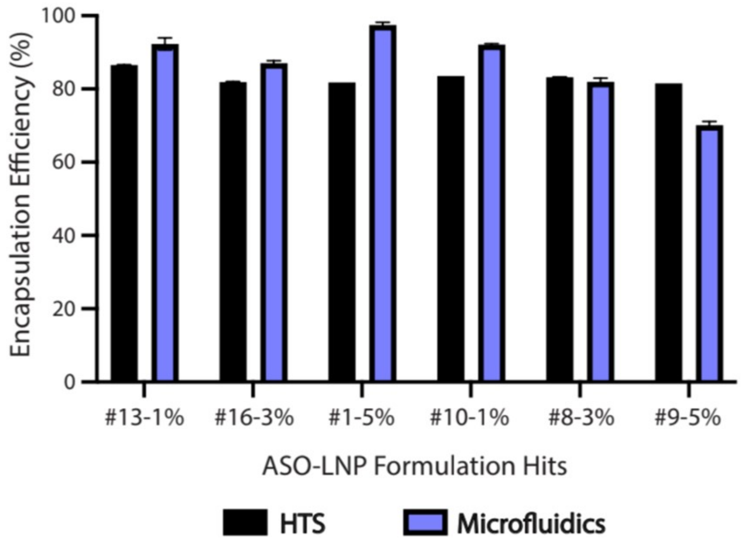

Figure S3. Encapsulation efficiencies are comparable across ASO-LNP hit formulations. Encapsulation efficiency was calculated based on total and free ASO amounts in each formulation, as determined using Oligreen assay and size exclusion chromatography, respectively.

Section S1. Regression analysis on LNP particle sizes:

A) Anionic PEG-lipids

- Linear Regression Model:
  *ASO-LNP hydrodynamic diameter (nm) = 225.71 - 0.44*carbon tail length(#C) - 0.05*PEG size (Da) - 14.12*PEG-lipid mol%*

- Coefficients:

	**Coefficients**	**Standard Error**	**t Stat**	**p-value**
Intercept	225.7093718	26.01575913	8.67587106	6.4911E-10
C-tail length (#C)	-0.445909286	1.509467593	-0.2954083	0.76959034
PEG size (Da)	-0.047938375	0.004190871	-11.438761	7.6631E-13
PEG-lipid mol %	-14.12145396	1.568862698	-9.001077	2.788E-10

- Model summary:
  R Square = 0.869, Adjusted R Square = 0.857, Standard Error = 15.37, Observations = 36
  ANOVA: Significance $F = 3.16 \times 10^{-14}$

B) Neutral PEG-lipids

- Linear Regression Model:
  *ASO-LNP hydrodynamic diameter (nm) = 172.68 + 1.29*carbon tail length(#C) - 0.03*PEG size (Da) - 6.21*PEG-lipid mol%*

- Coefficients:

	**Coefficients**	**Standard Error**	**t Stat**	**p-value**
Intercept	172.6862673	17.81521134	9.693192182	1.3723E-07
C-tail length (#C)	1.294659428	1.101953097	1.174877072	0.25964028
PEG size (Da)	-0.031757849	0.006357037	-4.99569956	1.9607E-04
PEG-lipid mol %	-6.209444167	2.271734507	-2.733349407	1.6165E-02

- Model summary:
  R Square = 0.700, Adjusted R Square = 0.636, Standard Error = 15.74, Observations = 18
  ANOVA: Significance $F = 5.9 \times 10^{-4}$
